# Abdominal Obesity, Hepatic Steatosis, Oxidative Stress and Diastolic Dysfunction in Patients with Metabolic Dysfunction-Associated Steatotic Liver Disease

**DOI:** 10.3390/ijms27041968

**Published:** 2026-02-18

**Authors:** Luca Colangeli, Ilaria Milani, Maria Eugenia Parrotta, Susanna Longo, Alessandro Nucera, Massimo Federici, Simonetta Palleschi, Barbara Rossi, Alessandro Mantovani, Saverio Muscoli, Frida Leonetti, Danila Capoccia, Paolo Sbraccia, Valeria Guglielmi

**Affiliations:** 1 Obesity Medical Center, University Hospital Policlinico Tor Vergata, 00133 Rome, Italy; luca.colangeli@ptvonline.it (L.C.);; 2 Department of Systems Medicine, University of Rome Tor Vergata, 00133 Rome, Italy; 3 Department of Medico-Surgical Sciences and Biotechnologies, Faculty of Pharmacy and Medicine, University of Rome La Sapienza, 04100 Latina, Italy; ila.milani94@gmail.com (I.M.);; 4 Department of Systems Medicine, Center for Atherosclerosis, University of Rome Tor Vergata and Policlinico Tor Vergata, 00133 Rome, Italy; 5 Department of Environment and Health, Istituto Superiore di Sanità, Viale Regina Elena 299, 00161 Rome, Italy; 6 Department of Medicine, University of Verona, 37129 Verona, Italy; 7 Metabolic Diseases Research Unit, IRCCS Sacro Cuore-Don Calabria Hospital, 37024 Negrar di Valpolicella, Italy; 8 Division of Cardiology, University Hospital Policlinico Tor Vergata, 00133 Rome, Italy

**Keywords:** MASLD, obesity, central adiposity, diastolic dysfunction, fatty liver index, CAP, FibroScan, vibration-controlled transient elastography, oxidative stress

## Abstract

Metabolic dysfunction-associated steatotic liver disease (MASLD) is increasingly recognized as a key contributor to the development of heart failure with preserved ejection fraction in individuals with obesity. This study aimed to investigate whether MASLD and diastolic dysfunction are independently associated with abdominal obesity through shared metabolic and oxidative mechanisms. We conducted a cross-sectional study in a tertiary university hospital including patients aged ≥ 50 years with obesity and MASLD. Clinical, anthropometric, biochemical, and oxidative stress parameters were collected, and hepatic steatosis and fibrosis were assessed using vibration-controlled transient elastography (FibroScan^®^). Patients were stratified according to the presence or absence of echocardiographic diastolic dysfunction. A total of 73 patients was included in the analysis and 27.4% had diastolic dysfunction. Patients with diastolic dysfunction were older and had higher body weight, body mass index (BMI) and waist circumference. Markers of hepatic steatosis, including fatty liver index (FLI) and controlled attenuation parameter (CAP), were higher in patients with diastolic dysfunction, whereas fibrosis measures were not. CAP was independently associated with diastolic dysfunction after adjustment for age and sex, but this association was lost after further adjustment for waist circumference, suggesting a mediating role of central adiposity. Plasma glutathione was inversely associated with FLI, but oxidative stress markers were not associated with diastolic dysfunction or steatosis severity. In conclusion, in patients ≥ 50 years with MASLD and obesity, diastolic dysfunction was common and closely related to abdominal obesity, highlighting MASLD as a multisystem condition with early cardiac involvement.

## 1. Introduction

Obesity is a common and independent risk factor for heart failure (HF), irrespective of ejection fraction (EF). However, epidemiological evidence shows an especially strong and independent link between obesity and the development of heart failure with preserved ejection fraction (HFpEF) [1].

HFpEF is a complex and often underdiagnosed syndrome defined by the presence of symptoms and/or signs of HF, evidence of structural or functional cardiac abnormalities and preserved left ventricular ejection fraction (LVEF ≥ 50%) [2]. Its pathophysiology is largely driven by diastolic dysfunction, a functional impairment of left ventricular (LV) relaxation resulting from cardiovascular remodeling and myocardial fibrosis [3]. Nevertheless, the underlying mechanisms of diastolic dysfunction in obesity remain incompletely understood and are likely multifactorial, involving dysregulated adipokine signaling, chronic low-grade inflammation, increased epicardial adipose tissue and sodium retention [4]. Despite growing recognition, HFpEF is still too often overlooked in patients with obesity, resulting in undertreatment [5].

Another major condition linked to visceral adiposity is metabolic-associated steatotic liver disease (MASLD), the most common liver disease worldwide, affecting approximately 38% of the adult population [6]. As the name implies, MASLD is strongly associated with metabolic dysfunctions, particularly obesity and type 2 diabetes (T2D), with visceral adiposity representing the key underlying mechanism [7]. Subjects affected by MASLD are at increased risk of cardiovascular events, whose underlying multifactorial pathophysiology overlaps substantially with obesity-related HFpEF, involving chronic low-grade inflammation, increased reactive oxygen species (ROS), and adipokine dysregulation [8]. Excess fatty acid accumulation promotes systemic oxidative stress and reduces antioxidant defenses, impairing endothelial function by lowering vasodilator bioavailability and increasing endothelium-derived contractile factors [9].

Previous studies have identified biochemical markers of oxidative stress that characterize both obesity and hepatic steatosis and are also linked to cardiovascular disease. These include elevated homocysteine levels, increased reactive oxygen metabolites, and reduced antioxidant defenses such as glutathione depletion [10,11,12,13]. Several of these markers have also been associated with poorer prognosis in patients with HF [14,15,16,17].

Importantly, MASLD is also more frequently associated with HFpEF than with HFrEF [18]. Most studies have shown an association between elevated fibrosis-4 (FIB-4) scores and a higher prevalence of HFpEF, but only few have used vibration-controlled transient elastography to assess hepatic stiffness or steatosis in relation to diastolic dysfunction evaluated by echocardiography [18].

In this study, we aim to determine the prevalence of liver steatosis and fibrosis assessed by FibroScan^®^ and to evaluate their relationship with echocardiographic markers of diastolic dysfunction and oxidative stress levels in an outpatient population.

## 2. Results

### 2.1. Study Population and Variables Associated with Diastolic Dysfunction

A total of 102 patients with MASLD and obesity were initially enrolled. Three were excluded due to a history suggestive of ischemic heart disease, one for being younger than 50 years, one for an alcohol use disorders identification test (AUDIT) score > 8, two because steatosis was not confirmed by either vibration-controlled transient elastography (VCTE) or fatty liver index (FLI) score, and twenty-two due to inconclusive cardiac ultrasound data. Consequently, 73 patients were included in the final analysis. Patients’ characteristics are summarized in Table 1.

Twenty patients (27.4%) had echocardiographic evidence of diastolic dysfunction. These patients were older than those without (64.4 ± 4.0 vs. 61.4 ± 5.8 years, *p* < 0.05). No significant differences were observed in sex distribution, or in the prevalence of diabetes, hypertension, dyslipidemia, obstructive sleep apnea syndrome, atrial fibrillation, hypothyroidism, smoking status, or use of antihypertensive, antihyperlipidemic, or antidiabetic medications.

Patients with diastolic dysfunction had significantly higher body weight (109.1 ± 15.6 vs. 93.9 ± 15.0 kg, *p* < 0.001), body mass index (BMI, 37.1 ± 4.3 vs. 34.4 ± 4.5 kg/m^2^, *p* < 0.05), and waist circumference (WC, 122.3 ± 9.8 vs. 113.1 ± 9.8 cm, *p* < 0.01). Heart rate, blood pressure, biochemical parameters and oxidative stress markers did not differ significantly between groups. Hepatic assessments showed higher FLI values (95.6 [90.0–98.3] vs. 88.3 [69.4–95.9], *p* < 0.05) and controlled attenuation parameter (CAP) measurements (300.0 ± 50.7 vs. 266.8 ± 52.2 dB/m, *p* < 0.05) compared to patients without diastolic dysfunction. No significant differences were observed in fibrosis markers, including the fibrosis-4 (FIB-4) score and liver stiffness measurement (LSM).

In multivariate logistic regression analyses adjusted for age and sex (Table 2), body weight (OR_adj_ 1.087, 95% CI 1.040 to 1.358; *p* < 0.001), BMI (OR_adj_ 1.173, 95% CI 1.034 to 1.331; *p* < 0.05), and WC (OR_adj_ 1.072, 95% CI 1.016 to 1.132; *p* < 0.05) were independently associated with diastolic dysfunction. Among hepatic steatosis measures, CAP was significantly associated with diastolic dysfunction (OR_adj_ 1.012, 95% CI 1.001 to 1.024; *p* < 0.05), whereas FLI showed a positive but non-significant association.

In a multivariate logistic regression model assessing the independent association between CAP and diastolic dysfunction after adjustment for age, sex and WC, CAP was not significantly associated with diastolic dysfunction (OR 1.007, 95% CI 0.993 to 1.020; *p* = 0.328). These results suggest that the apparent association between CAP and diastolic dysfunction is largely mediated by abdominal adiposity, as reflected by WC.

### 2.2. Evaluation of Echocardiographic Parameters

A The multivariate linear regression analysis assessing the association between MASLD clinical variables and all echocardiographic parameters considered in this study, adjusted for age and sex, is shown in Supplementary Table S1.

CAP showed a modest but significant positive association with several left ventricle structural parameters, including interventricular septum wall thickness in diastole (SIVd, B_adj_ = 0.001, 95% CI 0.000 to 0.002; *p* = 0.022), interventricular posterior wall thickness in diastole (PPd, Badj = 0.001, 95% CI 0.000 to 0.002; *p* = 0.008), left ventricular mass index (LVMi, B_adj_ = 0.106, 95% CI 0.017 to 0.196; *p* = 0.021), relative wall thickness (RWT, B_adj_ = 0.001, 95% CI 0.000 to 0.001; *p* = 0.020), and tricuspid regurgitation peak velocity (B_adj_ = 0.017, 95% CI 0.002 to 0.033; *p* = 0.031). No significant associations were observed between CAP and diastolic function indices (E wave, A wave, E/A ratio, and E/e′ ratios).

Similarly, FLI was positively associated with SIVd (B_adj_ = 0.005, 95% CI 0.002 to 0.009; *p* = 0.007) and negatively associated with septal E/e′ ratio (B_adj_ = −0.064, 95% CI −0.016 to −0.012; *p* = 0.018) and lateral E/e′ ratio (B_adj_ = −0.058, 95% CI −0.108 to −0.007; *p* = 0.026), indicating a subtle relationship with diastolic function.

WC and BMI showed stronger associations with left ventricular structural parameters, including SIVd, PPd, left ventricular end-diastolic diameter (DTD), and LVMi, and BMI was additionally associated with A wave velocity (B_adj_ = 2.154, 95% CI 0.264 to 4.044; *p* = 0.029), supporting a role of abdominal adiposity as potential mediator of the observed cardiac remodeling.

Other non-invasive markers of liver fibrosis, including LSM and FIB-4, showed mostly non-significant associations with cardiac structure and function, with the exception of associations between FIB-4 and E wave (B_adj_ = 1.609, 95% CI 0.583 to 2.636; *p* = 0.005) and SIVd (B_adj_ = −0.012, 95% CI −0.022 to −0.002; *p* = 0.025).

**Figure 1:**
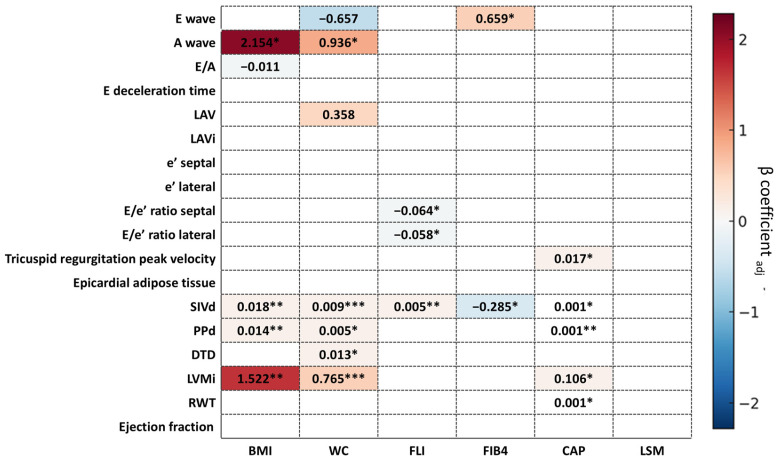
Heatmap of significant associations between clinical and echocardiographic parameters. Multivariable linear regression analysis. Values represent β_adj_ coefficient (age- and gender-adjusted standard regression coefficient). * *p*-value < 0.05, ** *p*-value < 0.01, *** *p*-value < 0.001. Abbreviations: A wave, Atrial contraction transmitral flow velocity; BMI, Body Mass Index; CAP, Controlled Attenuation Parameter; DTD, Left ventricular end-diastolic diameter; E/A, Ratio of E wave to A wave velocity; E deceleration time, Early diastolic deceleration time; E/e′ ratio lateral, Ratio of E wave to lateral e′; E/e′ ratio septal, Ratio of E wave to septal e′; E wave, Early diastolic transmitral flow velocity; Ejection Fraction, Left ventricular ejection fraction; Epicardial adipose tissue, Epicardial fat thickness; FIB4, fibrosis 4 index; FLI, Fatty Liver Index; LAV, Left atrial volume; LAVi, Left atrial volume index; LSM, liver stiffness measurement; LVMi, Left ventricular mass index; PPd, Posterior wall thickness in diastole; RWT, Relative wall thickness; SIVd, Interventricular septum thickness in diastole; Tricuspid regurgitation peak velocity, Peak velocity of tricuspid regurgitant jet; WC, waist circumference; e′ lateral, Lateral mitral annular early diastolic velocity; e′ septal, Septal mitral annular early diastolic velocity.

Overall, these findings suggest that hepatic steatosis (as assessed by CAP and FLI) shows modest associations with structural cardiac changes, whereas adiposity, particularly WC and BMI, emerges as the primary determinant of left ventricular remodeling patterns linked to diastolic dysfunction in this cohort (Figure 1).

### 2.3. Oxidative Stress

In age- and gender-adjusted linear regression analyses (Supplementary Table S2), both WC and BMI showed selective associations with oxidative stress indices. WC was positively associated with derivatives of reactive oxygen metabolites (dROMs, B = 1.405, 95% CI 0.143 to 2.666; *p* = 0.030) whereas BMI showed significant inverse associations with total free thiols (SH) levels (B = −2.681, 95% CI −4.734 to −0.628; *p* = 0.011) and SH-to-protein ratio (SHp, B = −0.048, 95% CI −0.080 to −0.017; *p* = 0.003), indicating increased lipid peroxidation and altered protein-thiol redox status with increasing adiposity. No significant associations were found for advance oxidation protein products (AOPP) or glutathione-related metabolites (cysteine, cysteinylglycine and glutathione).

In multivariable models adjusted for age and sex, oxidative stress markers showed limited associations with indices of hepatic steatosis and fibrosis (Supplementary Table S3). Among the biomarkers examined, only glutathione demonstrated a significant inverse relationship with FLI (B = −4.126, 95% CI −8.071 to −0.182; *p* < 0.05), indicating that higher hepatic steatosis was associated with lower glutathione levels. This association remained significant even after further adjustment for smoking status (B_adj_ = −4.176; 95% CI −8.175 to −0.176; *p* < 0.05). No other oxidative stress markers, including AOPP, SH, dROMs, cysteine, or cysteinylglycine, showed significant associations with FLI. Similarly, none of the oxidative stress parameters were significantly associated with FIB4, CAP, or LSM.

To further investigate this, participants were stratified by CAP into stage 0 versus stage 3 steatosis, and oxidative stress markers were compared between these subgroups. Consistent with the overall analysis, no statistically significant differences were found even at these extremes of hepatic steatosis (Table 3).

## 3. Discussion

In our cohort of patients with obesity and MASLD aged over 50 years, diastolic dysfunction was common, affecting 27.4% of individuals. This highlights its clinical relevance, as many patients remain asymptomatic despite subclinical cardiac impairment. Diastolic dysfunction often precedes overt HFpEF [19], and its detection is challenging because frequently symptoms overlap with those of obesity [1]. Our findings suggest that noninvasive assessment of hepatic steatosis may serve as a useful tool for identifying individuals at increased risk of cardiac dysfunction.

Consistent with emerging evidence linking obesity and MASLD to HFpEF phenotype (characterized by increased LV stiffness, impaired relaxation, and early concentric remodeling driven by adipose tissue inflammation) [1,20], advancing age, higher BMI, and increased WC were the primary determinants of diastolic dysfunction, along with hepatic steatosis indices, particularly CAP and FLI. Higher BMI and WC were also associated with greater SIVd and PPd thickness and with increased LVMi. Although concentric LVH in obesity-related HFpEF is typically defined by increased LVMi and RWT [21,22,23], we did not observe a correlation between BMI, WC and RWT. However, CAP was significantly associated with both LVMi and RWT, indicating a potential link between liver steatosis and concentric remodeling, consistent with previous findings that severe MASLD correlates with concentric left ventricular hypertrophy (LVH) [24].

In contrast, in our study, FLI was not correlated with LVMi, but was associated with SIVd thickness and with E/e′ and showed a trend with E/A ratio, consistent with prior reports [25,26,27]. Previous studies have also described associations between FLI and RWT [28], LVMi [29], and LV global longitudinal strain [25]. Collectively, these findings support a link between FLI, cardiac remodeling and diastolic dysfunction, although obesity was underrepresented in the populations studied.

Diagnostic accuracy of FLI to identify steatosis is suboptimal in women after menopause compared to men [30], and, in our study, we had a slightly higher prevalence of males in diastolic dysfunction group. In our multivariate models adjusted for age and sex, only CAP remained independently associated with diastolic dysfunction, while FLI showed a non-significant trend. However, after further adjustment for WC, the association between CAP and diastolic dysfunction was no longer significant. Rather than representing a negative finding, this result underscores the central role of abdominal adiposity in the pathophysiological link between hepatic steatosis and cardiac remodeling. The attenuation of the association after inclusion of WC suggests that central adiposity may largely account for the relationship between liver fat and diastolic dysfunction. Unexpectedly, no significant differences were observed for the waist-to-height ratio (WHtR), likely due to uniformly high values in our cohort (mean 0.69 ± 0.07). In the PARAGON-HF trial, which randomized 4796 patients with HFpEF, nearly all participants had a WHtR ≥ 0.5 and, among those with a BMI < 30 kg/m^2^, almost 40% had a WHtR ≥ 0.6 [31]. These findings support WHtR as an important contributor to LV structural changes, although it is likely insufficient on its own to cause overt diastolic dysfunction.

The development of diastolic impairment likely requires additional factors and may depend on both the duration and severity of abdominal obesity, but not aging per se. Although our study identified an association between age and the development of diastolic dysfunction in patients with MASLD, age does not appear to be the most important factor. Previous studies have shown that even children with MASLD develop diastolic dysfunction [32]. Consistent with our findings, we hypothesize that central adiposity may account for this increased risk in younger patients with MASLD. Abdominal obesity may act as the primum driver of cardiac structural changes, while metabolic complications such as hypertension, T2D, and hepatic steatosis further amplify this risk. In patients with T2D, MASLD has been associated with lower E/A ratios, higher E/e′ ratios, and increased LVMi [33]. Similarly, in hypertensive populations, liver steatosis has been linked to both eccentric and concentric remodeling as well as to diastolic dysfunction [34]. Importantly, diastolic dysfunction and cardiac remodeling also create a substrate for arrhythmias, and previous studies reported a higher incidence of atrial fibrillation [35] and ventricular arrhythmias [36] in MASLD. At the same time, increased secretion of pro-inflammatory adipokines and hepatokines, along with macrophage infiltration, amplifies both hepatic and systemic inflammatory responses, further impairing insulin signaling and accelerating MASLD progression [37]. Systemic inflammation, in turn, plays a central role in the development of adverse cardiac remodeling by inducing the expression of adhesion molecules and facilitating the recruitment of immune cells into the myocardium [38,39]. Pro-inflammatory cytokines, including interleukin-1 (IL-1), interleukin-6 (IL-6), and tumor necrosis factor- α (TNF-α), contribute to myocardial remodeling by disrupting calcium homeostasis and intercellular communication and by promoting myolysis, cardiomyocyte apoptosis, and myocardial fibrosis [38,40]. Together, these findings, which align with our results, suggest a continuum connecting visceral adiposity, hepatic steatosis, diastolic dysfunction and arrhythmogenesis, highlighting an additional clinical consequence of MASLD beyond diastolic dysfunction. Cardiac hypertrophy is often accompanied by myocardial fibrosis, which increases stiffness and impairs diastolic function [5]. Both myocardial and hepatic fibrosis likely develop over the course of long-standing obesity through chronic low-grade inflammation [1]. Although liver biopsy remains the gold standard for the diagnosis of hepatic fibrosis, employing FIB-4 for preliminary risk assessment and subsequently applying VCTE offers a practical and reliable pathway for fibrosis evaluation in routine clinical care [41]. In our cohort, however, no correlation was observed between noninvasive liver fibrosis markers (FIB-4 and LSM) and cardiac remodeling, likely because most patients were classified as F0 on FibroScan and few had advanced fibrosis. Previous studies have reported stronger associations at higher fibrosis stages [42,43]. Notably, FIB-4 has also been shown to predict adverse outcomes in HF populations, particularly in HFpEF [44,45,46,47].

Collectively, these findings suggest that in MASLD, visceral adiposity and hepatic steatosis contribute to early left ventricular remodeling, even in the absence of advanced hepatic fibrosis. While hepatic and myocardial fibrosis represent the structural endpoint of chronic metabolic injury, the cascade leading to diastolic dysfunction likely begins much earlier, driven by systemic metabolic stress.

Against this background, we sought to determine whether oxidative stress might constitute an early player linking MASLD to diastolic dysfunction. In obesity, oxidative stress reflects a cluster of systemic clinical and metabolic disturbances linked to adipose tissue dysfunction [48], and higher body fat is consistently associated with impaired antioxidant capacity and increased oxidative injury to proteins and lipids [49,50,51], together with alterations in plasma redox status, including thiol and disulfide levels [52,53]. In our study, BMI was positively associated with altered plasma redox status—reflected by lower SH levels and a reduced SH/protein ratio—whereas WC correlated positively with lipid peroxidation (dROMs). These results suggest that BMI and WC provide complementary information, with BMI more accurately reflecting the overall metabolic burden of adiposity on systemic redox balance, and WC specifically capturing the contribution of visceral fat to oxidative stress. In contrast, no significant correlation was observed between BMI or WC and protein oxidative damage (AOPP), a result possibly due to the relatively high AOPP values measured in our cohort [48]. However, aside from the inverse association between glutathione and FLI, in our cohort we found no significant relationships between circulating oxidative stress markers and liver parameters, and no differences in oxidative stress marker levels between participants with and without diastolic dysfunction. These largely neutral findings should be interpreted with caution. Circulating redox biomarkers may not adequately reflect tissue-specific oxidative stress within the liver or myocardium, where local mitochondrial dysfunction, lipid accumulation, and inflammatory signaling can generate compartmentalized oxidative injury not captured in peripheral blood measurements [54]. Therefore, the absence of systemic redox alterations does not exclude the presence of organ-level oxidative stress contributing to hepatic or cardiac remodeling. Visceral obesity is a major determinant of systemic insulin resistance and plays a central role in increasing free fatty acid (FFA) flux to the liver. In parallel, hyperinsulinemia and excess glucose stimulate hepatic de novo lipogenesis, further contributing to intrahepatic lipid accumulation [55]. Lipid overload therefore places particular metabolic strain on the liver [9]. Within this pathophysiological context, peroxisome proliferator-activated receptors (PPARs) critically regulate hepatic lipid homeostasis [56]. PPAR-α enhances fatty acid β-oxidation and mitigates lipotoxicity, whereas PPAR-γ modulates adipose tissue lipid storage and systemic insulin sensitivity, indirectly influencing hepatic FFA delivery. Impaired PPAR signaling in visceral obesity may therefore exacerbate defective lipid handling, oxidative stress, and inflammatory activation, thereby fostering disease progression. Thus, lipotoxicity represents the first hit in the multiple-hit model, triggering excess ROS generation and endoplasmic reticulum stress [57] and increasing hepatic vulnerability to subsequent metabolic insults (the second hit), which amplify lipotoxicity, oxidative stress and inflammation and drive the development of MASLD and progression to MASH [9,58]. In this framework, the absence of an association between oxidative stress markers and CAP in our cohort, even when comparing individuals without steatosis (S0) to those with severe steatosis (S3), become plausible. CAP quantifies liver fat content alone but may not capture systemic metabolic dysfunction. In contrast, FLI integrates BMI, waist circumference, triglycerides, and gamma-glutamyl transferase (GGT), thereby reflecting not only hepatic involvement but a systemic metabolic impairment [59]. The inverse association between FLI and glutathione in our study aligns with consistently reduced glutathione levels reported in MASLD [60,61]. Mitochondria are central to cellular energy production through the respiratory chain but also constitute a major source of ROS. To maintain redox homeostasis, glutathione serves as the primary defense against oxidative damage [62]. Impaired mitochondrial redox balance exposes hepatocytes to chronic inflammation, necrosis, and apoptosis, thereby promoting progression to metabolic dysfunction-associated steatohepatitis (MASH) [62,63]. These mechanisms support the therapeutic rationale for glutathione supplementation, which has shown beneficial effects in MASLD patients [62]. Moreover, our findings are consistent with prior evidence showing no correlation between oxidative stress markers such as dROMs and HFpEF [64]. Similarly, Negi et al. reported no differences in circulating redox markers, including dROMs and glutathione/cysteine ratios, between patients with and without HFpEF or diastolic dysfunction, suggesting that ROS signaling is compartmentalized and that localized myocardial oxidative stress may be sufficient to induce diastolic dysfunction [54]. Further studies are needed to determine whether systemic redox biomarkers can serve as early non-invasive indicators of cardiac remodeling.

This study has several limitations. First, cohort included only individuals with obesity, MASLD, and an age > 50 years; inclusion of a comparison group (e.g., MASLD with BMI < 30 kg/m^2^) would have clarified the relative contributions of obesity and MASLD to diastolic dysfunction. Second, the uniformly elevated WHtR prevented evaluation of its discriminative value for cardiovascular risk stratification [65]. Third, in individuals with obesity, a proportion of diastolic evaluations typically falls into the “inconclusive” category of the ASE/EACVI algorithm due to limitations in Doppler acquisition. In this regard, the acoustic window limitations typical of patients with obesity may have affected the acquisition of key diastolic parameters, particularly tissue Doppler velocities and TR velocity, potentially leading to more inconclusive assessments or reduced sensitivity to detect early diastolic abnormalities. Furthermore, E/e′ ratios may underestimate filling pressures in individuals with severe obesity due to increased annular excursion, potentially attenuating differences between groups. Importantly, the exclusion of a substantial proportion of patients with inconclusive diastolic assessment represents a potential source of selection bias, as individuals with technically limited studies may differ systematically in terms of body habitus or cardiac remodeling. Finally, the modest sample size limited our ability to assess the independent effects of key covariates, such as hypertension, that strongly influence diastolic dysfunction. In addition, the focus on a cohort of older adults with obesity and MASLD restricts the generalizability of our findings and precludes separating the distinct contributions of obesity and MASLD to cardiac remodeling.

## 4. Materials and Methods

### 4.1. Study Design, Participants and Clinical Assessment

An observational study was conducted in patients aged ≥ 50 years diagnosed with MASLD [7] and obesity (defined as body mass index or BMI ≥ 30 kg/m^2^) at the Obesity Medical Center of the Policlinico Tor Vergata University Hospital in Rome, Italy, from September 2024 to April 2025.

Exclusion criteria were as follows: (1) age younger than 50 years; (2) concomitant chronic liver diseases (e.g., viral, drug-induced liver injury, autoimmune hepatitis, hemochromatosis, primary sclerosing cholangitis, primary biliary cholangitis); (3) presence of hepatocellular carcinoma or other malignancies; (4) excessive alcohol intake, defined as AUDIT score ≥ 8 (see below); (5) history of ischemic heart disease; (6) New York Heart Association (NYHA) class IV heart failure [66] or recent (<90 days) hospitalization for heart failure; (7) pregnancy or breastfeeding.

The enrolled subjects underwent a detailed medical history assessment including age (years), sex, T2D, obstructive sleep apnea syndrome (OSAS), atrial fibrillation and hypothyroidism diagnosis, use of antihypertensive or antihyperlipidemic medications and cigarette smoking status (never, former or current). T2D was defined as fasting plasma glucose ≥ 126 mg/dL, 2 h plasma glucose ≥ 200 mg/dL after a 75 g oral glucose tolerance test, HbA1c ≥ 6.5%, clinical diagnosis by a clinician, and/or use of antidiabetic medication [67]. Hypertension was defined as systolic blood pressure ≥ 140 mmHg and/or diastolic blood pressure ≥ 90 mmHg or current use of antihypertensive medication. Dyslipidemia was defined as self-reported use of lipid-lowering medications or at least one abnormality in lipid markers including hypercholesterolemia and/or hypertriglyceridemia and/or hyper-LDL and/or hypo-HDL [68].

Alcohol consumption was assessed using the validated AUDIT questionnaire. A score ≥ 8 was considered indicative of excessive alcohol intake [69].

Height and weight were measured with participants barefoot and wearing light clothing using a calibrated scale equipped with a standard stadiometer.WC was measured at the level of the umbilicus with the patient standing upright, using a non-elastic anthropometric tape. BMI was calculated as weight in kilograms divided by the square of height in meters (kg/m^2^). WHtR was calculated by dividing WC by height.

Heart rate, systolic blood pressure (SBP) and diastolic blood pressure (DPB) were recorded at rest with participants seated, using a manual sphygmomanometer and a stethoscope, according to standard clinical procedures.

Venous blood samples were collected in the morning from fasting subjects and analyzed immediately.

Laboratory assessments included complete blood count and measurements of fasting plasma glucose (FPG, mg/dL), glycosylated hemoglobin (HbA1c, mmol/mol), total cholesterol (TC, mg/dL), high-density lipoprotein cholesterol (HDL-C, mg/dL), triglycerides (TG, mg/dL), C-reactive protein (CRP, mg/L), serum creatinine (Cr, mg/dL), alanine aminotransferase (ALT, IU/L), aspartate aminotransferase (AST, IU/L), GGT (IU/L), homocysteine (µmol/L). Friedewald equation was used to assess low-density lipoprotein cholesterol (LDL-C). All biochemical analyses were performed in a certified clinical laboratory using standardized methods and according to the manufacturer’s instructions.

### 4.2. Oxidative Stress Assessment

Plasma samples were separated within 2 h of blood collection and immediately stored at −80 °C until analysis. Oxidative stress was evaluated by measuring plasma biomarkers of oxidative damage to proteins (AOPP) and lipids (dROMs). Redox status was assessed by quantifying SH and SHp. Markers of glutathione metabolism included total glutathione, cysteine, and cysteinylglycine. dROMs were measured using the d-ROMs test (Diacron, Grosseto, Italy) according to the manufacturer’s instructions. All other analytes were quantified by colorimetric assays or by high-performance liquid chromatography with fluorescence detection, as previously described [48].

### 4.3. Hepatic Assessment

MASLD was diagnosed in patients with evidence of hepatic steatosis detected by VCTE (defined by a CAP ≥ 248 dB/min) or a FLI score ≥ 60 [7], in the presence of at least one cardiometabolic risk factor and in the absence of excessive alcohol intake (defined as intake of >210 g/week for men and >140 g/week for women) or other liver diseases, in accordance with recent clinical recommendations [7].

The FLI was calculated using the following formula [70]:

FLI = [e^(0.953 × ln(TG) + 0.139 × BMI + 0.718 × ln(GGT) + 0.053 × WC − 15.745)]/[1 + e^(0.953 × ln(TG) + 0.139 × BMI + 0.718 × ln(GGT) + 0.053 × WC − 15.745)] × 100.

The FIB-4 index was calculated using the following formula [7]:

FIB-4 = age [years] × AST [U/L]/(platelet count [109/L] × √(ALT [U/L])).

All participants underwent VCTE with FibroScan^®^ after an overnight fast. With the patient in the supine position and the right arm abducted, the ultrasound probe was placed on the skin above the right mid-axillary line. The median of at least 10 valid measurements was used to represent the LSM (kPa). Only measurements with an interquartile range (IQR) < 30% of the median liver stiffness value were considered valid. Hepatic steatosis was simultaneously assessed by CAP (dB/m). Grades of steatosis were defined consistently with established guidelines [7,71] as follows:

- S0 (no steatosis): <248 dB/m.
- S1 (mild): ≥248 dB/min and <268 dB/m.
- S2 (moderate): ≥268 dB/min and <280 dB/m.
- S3 (severe): ≥280 dB/m.

Fibrosis staging was based on the following LSM cut-offs [7,72]:

- F0: <7.9 kPa.
- F1 (significant): ≥7.9 kPa and <9.5 kPa.
- F2 (advanced): ≥9.5 kPa and <12.5 kPa.
- F3-4 (cirrhosis): ≥12.5 kPa.

### 4.4. Echocardiographic Assessment

Two physicians experienced in echocardiographic assessment performed comprehensive transthoracic echocardiography using a Samsung HS40 ultrasound system (Samsung Medison Co., Ltd, Seoul, Republic of Korea) equipped with an H8-4P CW probe. Standard two-dimensional and Doppler measurements included early (E) and late (A) diastolic transmitral flow velocities, E/A ratio, early diastolic deceleration time, left atrial volume (LAV) and volume index (LAVi), septal and lateral mitral annular early diastolic velocities (e′ septal and e′ lateral) measured using Tissue Doppler Imaging in the apical four-chamber view, corresponding E/e′ ratios, tricuspid regurgitation peak velocity, epicardial adipose tissue thickness, SIVd and PPd, DTD, RWT, and LVEF using Simpson’s rule, according to current guidelines [73]. LVMi was automatically calculated by the echocardiograph according to the ASE/EACVI recommendations [73].

Diastolic dysfunction was assessed according to the 2016 ASE/EACVI recommendations [2]. Patients with LVEF < 50% were excluded. The diagnostic evaluation was based on the recommended variables: septal e′ velocity < 7 cm/s, lateral e′ velocity < 10 cm/s, average E/e′ ratio > 14, LAVi > 34 mL/m^2^, and peak TR velocity > 2.8 m/s. LV diastolic function was classified as normal if ≤50% of available parameters were abnormal, as diastolic dysfunction if >50% were abnormal, and inconclusive if results were equivocal.LVH was defined as LVMI > 115 g/m^2^ in men and >95 g/m^2^ in women [73]. In patients with LVH, diastolic function grading followed the guideline algorithm incorporating the E/A ratio and, when required, additional parameters (E velocity, TR velocity, E/e′ ratio, and LAVi).

### 4.5. Statistical Analysis

This cross-sectional study was designed as a pilot investigation; consequently, a formal sample size calculation was not undertaken. For descriptive statistical analysis, categorical variables were expressed as frequencies and percentages and continuous variables were expressed as mean ± standard deviation (SD) for normally distributed data or as median and interquartile range (IQR) for non-normally distributed data. The Shapiro–Wilk test was used to assess the normality of the data distribution. Group comparisons were performed using Student’s *t*-test, Mann–Whitney U test or chi-squared test, as appropriate.

Multivariate logistic regression was performed to assess age- and gender-adjusted odds ratios for the associations between clinical variables and diastolic dysfunction. Linear regression analysis, adjusted for age and gender, was used to estimate regression coefficients for the associations between clinical variables and echocardiographic parameters and for the associations between clinical variables and oxidative stress markers.

A *p*-value < 0.05 was considered as significant. All analyses were conducted using IBM SPSS Statistics for Windows, Version 23.0 (IBM Corp., Armonk, NY, USA).

## 5. Conclusions

In conclusion, in patients over 50 with obesity and MASLD, diastolic dysfunction was common and closely linked to central adiposity and hepatic steatosis. Visceral fat appears to drive early left ventricular remodeling, while hepatic steatosis may amplify this risk through shared metabolic and inflammatory pathways. Systemic oxidative stress mostly reflected metabolic dysfunction rather than liver disease and, although it did not correlate with echocardiographic parameters, its association with adiposity underscores its role in the metabolic milieu contributing to both hepatic and myocardial alterations.

These findings support MASLD as a multisystem disease with subclinical cardiac involvement, even in the absence of overt heart failure or advanced fibrosis. Noninvasive assessment of hepatic steatosis may help identify patients at risk of early cardiac dysfunction, who might benefit from targeted lifestyle and pharmacologic interventions aimed at reducing visceral adiposity and metabolic inflammation. Larger prospective studies are needed to clarify the links between hepatic steatosis, oxidative stress, and diastolic impairment, and to evaluate the potential benefit of integrated metabolic–cardiac screening in MASLD.

## Figures and Tables

**Table 1:** Patients’ characteristics and comparisons between diastolic dysfunction groups.

	Total Population (*n* = 73)	Diastolic Dysfunction Yes (*n* = 20)	Diastolic Dysfunction No (*n* = 53)	*p*-Value
Age (years)	62.2 ± 5.5	64.4 ± 4.0	61.4 ± 5.8	**0.035**
Males (%)	39.7%	55.0%	34.0%	0.101
T2D (%)	24.7%	30.0%	22.6%	0.515
Hypertension (%)	68.5%	80.0%	64.2%	0.194
Dyslipidemia (%)	65.8%	70.0%	64.2%	0.639
OSAS (%)	9.6%	15%	7.5%	0.335
Atrial fibrillation (%)	5.6%	10.0%	3.8%	0.307
Hypotyroidism (%)	31.5%	25.0%	34.0%	0.462
Antihyperlidemic drugs (%)	58.9%	65.0%	56.6%	0.516
Antihypertensive drugs (%)	68.5%	80.0%	64.2%	0.194
Metformin (%)	29.2%	20.0%	32.7%	0.289
GLP-1 RA (%)	23.6%	30.0%	21.2%	0.429
SGLT2-i (%)	8.3%	15.0%	5.8%	0.204
Previous bariatric surgery (%)	6.8%	5.0%	7.5%	0.701
Smokers (%)	12.3%	25.0%	7.5%	0.127
AUDIT	1.0 [0.0–2.0]	1.0 [0.0–2.0]	1.0 [0.0–2.0]	0.453
Weight (kg)	98.12 ± 16.5	109.1 ± 15.6	93.9 ± 15.0	**<0.001**
BMI (kg/m^2^)	35.1 ± 4.6	37.1 ± 4.3	34.4 ± 4.5	**0.023**
Waist circumference (cm)	115.6 ± 12.0	122.3 ± 9.8	113.1 ± 9.8	**0.003**
WHtR	0.69 ± 0.07	0.71 ± 0.06	0.69 ± 0.08	0.150
Systolic blood pressure (mmHg)	120 [115–140]	130 [114–140]	120 [115–130]	0.494
Dyastolic blood pressure (mmHg)	70 [70–80]	70 [70–81]	70 [70–80]	0.731
Heart Rate (bpm)	72 ± 11	71 ± 11	72 ± 11	0.656
Fasting blood glucose (mg/dL)	98.7 ± 17.6	101.3 ± 11.6	97.5 ± 19.8	0.452
Hb1Ac (mmol/mol)	39.5 [36.0–42.0]	39.0 [36.0–42.0]	40.0 [36.0–42.0]	0.977
Total cholesterol (mg/dL)	185.1 ± 46.9	172.3 ± 40.9	190.3 ± 48.6	0.171
LDL–cholesterol (mg/dL)	93.0 ± 54.4	81.2 ± 50.9	97.5 ± 55.8	0.258
HDL–cholesterol (mg/dL)	51.4 [42.0–57.0]	50.0 [39.0–56.0]	52.0 [42.8–59.0]	0.299
Tryglicerides (mg/dL)	117.0 [93.5–145.0]	125.5 [109.3–180.8]	113.0 [90.0–142.3]	0.193
C-reactive protein (mg/L)	4.5 ± 5.4	3.8 ± 1.3	4.8 ± 6.5	0.383
Creatinine (mg/dL)	0.8 ± 0.2	0.9 ± 0.2	0.8 ± 0.2	0.170
ALT (UI/L)	25.0 [19.0–31.0]	25.3 [19.3–31.5]	24.0 [19.0–31.0]	0.536
AST (UI/L)	22.0 [19.0–25.8]	22.0 [20.0–25.5]	22.0 [18.0–25.5]	0.479
GGT (U/L)	24.0 [17.0–35.0]	27.5 [23.0–34.0]	23.0 [16.5–35.0]	0.252
Homocysteine (μmol/L)	12.4 [10.3–15.3]	12.3 [10.1–14.7]	12.4 [10.8–15.6]	0.374
AOPP (μmol/L)	56.3 [48.7–75.9]	57.6 [48.7–83.2]	55.8 [48.7–75.1]	0.494
SH (μmol/L)	478.5 ± 44.9	467.0 ± 43.4	483.0 ± 45.1	0.179
Proteins (g/dL)	6.9 ± 0.4	6.8 ± 0.4	7.0 ± 0.4	0.129
SH/p (μmol/g)	6.9 ± 0.7	6.9 ± 0.7	6.9 ± 0.7	0.649
dROMs (U.Carr)	415.1 ± 73.1	410.1 ± 46.8	417.0 ± 81.3	0.724
Glutathione (μmol/L)	4.6 ± 1.6	4.4 ± 1.2	4.6 ± 1.7	0.675
Cysteine (μmol/L)	333.7 ± 32.4	339.2 ± 38.4	331.7 ± 30.0	0.382
Cysteinylglycine (μmol/L)	32.4 [29.4–37.1]	32.4 [29.6–33.4]	32.4 [29.4–37.5]	0.493
FLI	93.1 [77.6–96.7]	95.6 [90.0–98.3]	88.3 [69.4–95.9]	**0.016**
FIB-4	1.2 ± 0.4	1.3 ± 0.4	1.2 ± 0.4	0.440
CAP (dB/m)	275.9 ± 53.5	300.0 ± 50.7	266.8 ± 52.2	**0.017**
CAP S0 (%)	34.3%	20.0%	39.6%	0.115
CAP S1 (%)	9.6%	5.0%	11.3%	0.413
CAP S2 (%)	6.8%	10.0%	5.7%	0.513
CAP S3 (%)	49.3%	65.0%	43.4%	0.100
LSM (kPa)	6.0 ± 2.0	6.6 ± 2.0	5.8 ± 2.0	0.133
LSM F0 (%)	79.5%	75.0%	81.1%	0.563
LSM F1 (%)	16.4%	20.0%	15.1%	0.614
LSM F2 (%)	4.1%	5.0%	3.8%	0.814
LSM F3 (%)	0.0%	0.0%	0.0%	-
Epicardial adipose tissue (cm)	0.61 [0.42–0.81]	0.67 [0.61–0.73]	0.56 [0.40–0.92]	0.138

Data are expressed as mean ± standard deviation, median [interquartile range], or percentage (%). Student’s *t*-test, Mann–Whitney U test, and Chi-squared test were applied as appropriate. *p*-values < 0.05 were considered statistically significant and are shown in bold. Abbreviations: ALT, Alanine Aminotransferase; AST, Aspartate Aminotransferase; AST/ALT, Alanine Aminotransferase to Aspartate Aminotransferase ratio; AUDIT, Alcohol Use Disorders Identification Test; AOPP, advanced oxidation protein products; BMI, Body Mass Index; CAP, Controlled Attenuation Parameter; dROMs, derivatives of reactive oxygen metabolites; F, Fibrosis; FIB-4, Fibrosis-4 Index; FLI, Fatty Liver Index; GGT, Gamma-Glutamyl Transferase; GLP-1 RAs, GLP1-receptor agonists; Hb1Ac, Glycated Hemoglobin; HDL, High-Density Lipoprotein; LDL, Low-Density Lipoprotein; LSM, Liver Stiffness Measurement; OSAS, Obstructive Sleep Apnea Syndrome; S, Steatosis; SGLT2-i, SGLT2-inhibitors; SH, total free thiols; SH/p, total free thiols normalized to protein content; T2D, type 2 diabetes; WHtR, Waist-to-height ratio.

**Table 2:** Age- and gender-adjusted odds ratios of associations between clinical variables and diastolic dysfunction.

Variable	OR_adj_	95% CI	*p*-Value
Weight	1.087	1.040 to 1.358	**<0.001**
BMI	1.173	1.034 to 1.331	**0.013**
WC	1.072	1.016 to 1.132	**0.011**
FLI	1.054	0.994 to 1.117	0.077
CAP	1.012	1.001 to 1.024	**0.039**

Multivariable linear regression model. Abbreviations: 95% CI, 95% Confidence Intervals; BMI, Body Mass Index; CAP, Controlled Attenuation Parameter; FLI, Fatty Liver Index; OR_adj_, age- and gender-adjusted Odds Ratio; WC, waist circumference. *p* values < 0.05 are shown in bold.

**Table 3:** Oxidative stress markers in S0 and S3 steatosis categories defined by CAP.

	S0 (*n* = 25)	S3 (*n* = 35)	*p*-Value
AOPP (μmol/L)	65.0 ± 19.1	64.5 ± 22.5	0.859
SH (μmol/L)	471.3 ± 43.4	482.3 ± 46.0	0.316
Proteins (g/dL)	6.9 ± 0.4	6.9 ± 0.3	0.906
SH/p (μmol/g)	6.8 ± 0.7	6.9 ± 0.7	0.412
dROMs (U.Carr)	421.7 ± 75.2	416.8 ± 67.4	0.952
Glutathione (μmol/L)	4.4 ± 1.7	4.7 ± 1.4	0.484
Cysteine (μmol/L)	329.0 ± 36.0	336.3 ± 30.7	0.386
Cysteinylglycine (μmol/L)	32.9 ± 5.0	33.3 ± 5.7	0.703

Data are expressed as mean ± standard deviation. Student’s *t*-test was applied. *p*-values < 0.05 were considered statistically significant. Abbreviations: AOPP, advanced oxidation protein products; CAP, Controlled Attenuation Parameter; dROMs, derivatives of reactive oxygen metabolites; S, Steatosis; SH/p, total free thiols normalized to protein content.

## Data Availability

The raw data supporting the conclusions of this article will be made available by the authors on request.

## Supplementary Materials

The following supporting information can be downloaded at https://www.mdpi.com/article/10.3390/ijms27041968/s1.

## Abbreviations

The following abbreviations are used in this manuscript:

A	Late diastolic transmitral flow velocity
ALT	Alanine aminotransferase
AOPP	Advanced oxidation protein products
AST	Aspartate aminotransferase
AUDIT	Alcohol use disorders identification test
BMI	Body mass index
CAP	Controlled attenuation parameter
Cr	Serum creatinine
CRP	C-reactive protein
DBP	Diastolic blood pressure
dROMs	Derivatives of reactive oxygen metabolites
DTD	Left ventricular end-diastolic diameter
E	Early diastolic transmitral flow velocity
EF	Ejection fraction
e′ lateral	Lateral mitral annular early diastolic velocity
e′ septal	Septal mitral annular early diastolic velocity
FIB-4	Fibrosis-4
FLI	Fatty liver index
FPG	Fasting plasma glucose
GGT	Gamma-glutamyl transferase
HbA1c	Glycosylated hemoglobin
HDL-C	High-density lipoprotein cholesterol
HF	Heart failure
HFpEF	Heart failure with preserved ejection fraction
IQR	Interquartile range
LAV	Left atrial volume
LAVi	Left atrial volume index
LDL-C	Low-density lipoprotein cholesterol
LSM	Liver stiffness measurement
LV	Left ventricular
LVEF	Left ventricular ejection fraction
LVH	Left ventricular hypertrophy
LVMi	Left ventricular mass index
MASLD	Metabolic dysfunction-associated steatotic liver disease
NYHA	New York Heart Association
OSAS	Obstructive sleep apnea syndrome
PPd	Interventricular posterior wall thickness in diastole
ROS	Reactive oxygen species
RWT	Relative wall thickness
SBP	Systolic blood pressure
SD	Standard deviation
SH	Total free thiols
SHp	Total free thiols normalized to protein content
SIVd	Interventricular septum wall thickness in diastole
T2D	Type 2 diabetes
TC	Total cholesterol
TG	Triglycerides
VCTE	Vibration controlled transient elastography
WC	Waist circumference
WHtR	Waist-to-height ratio
