# Supplementary material for: Abdominal Obesity, Hepatic Steatosis, Oxidative Stress and Diastolic Dysfunction in Patients with Metabolic Dysfunction-Associated Steatotic Liver Disease
Source: Int J Mol Sci. 2026 Feb 18;27(4):1968. doi: 10.3390/ijms27041968 (PMC12940709; doi:10.3390/ijms27041968)
Supplement: Supplementary file 1 [file ijms-27-01968-s001.zip › ijms-4147060-supplementary.pdf]

Table S1. Age- and gender-adjusted linear regression coefficients of associations between clinical variables and echocardiographic parameters.

Dependent Variable	Independent Variable	B_{adj}	95% CI	p value
E wave	BMI	-0.931	-2.772 to 0.910	0.308
A wave	BMI	2.154	0.264 to 4.044	**0.029**
E/A	BMI	-0.011	-0.022 to 0.001	**0.069**
E deceleration time	BMI	0.446	-3.699 to 4.592	0.830
LAV	BMI	0.648	-0.343 to 1.639	0.196
LAVi	BMI	0.144	-0.377 to 0.605	0.645
e′ septal	BMI	0.002	-0.156 to 0.161	0.975
e′ lateral	BMI	-0.086	-0.247 to 0.075	0.288
E/e′ ratio septal	BMI	-0.058	-0.294 to 0.177	0.622
E/e′ ratio lateral	BMI	-0.006	-0.307 to 0.294	0.967
Tricuspid regurgitation peak velocity	BMI	0.120	-0.054 to 0.294	0.174
Epicardial adipose tissue	BMI	-0.038	-0.155 to 0.080	0.526
SIVd	BMI	0.018	0.007 to 0.028	**0.001**
PPd	BMI	0.014	0.004 to 0.023	**0.007**
DTD	BMI	0.027	-0.003 to 0.057	**0.076**
LVMi	BMI	1.522	0.528 to 2.517	**0.003**
RWT	BMI	0.003	-0.002 to 0.008	0.176
Ejection Fraction	BMI	0.107	-0.198 to 0.412	0.487
E wave	WC	-0.637	-1.283 to 0.008	**0.053**
A wave	WC	0.936	0.280 to 1.592	**0.010**
E/A	WC	-0.004	-0.008 to 0.001	0.103
E deceleration time	WC	0.388	-1.231 to 2.007	0.634
LAV	WC	0.358	-0.036 to 0.752	0.074
LAVi	WC	0.108	-0.088 to 0.304	0.273
e′ septal	WC	0.002	-0.060 to 0.064	0.946
e′ lateral	WC	-0.038	-0.102 to 0.026	0.242
E/e′ ratio septal	WC	-0.022	-0.155 to 0.070	0.634
E/e′ ratio lateral	WC	0.016	-0.105 to 0.137	0.797
Tricuspid regurgitation peak velocity	WC	0.053	-0.015 to 0.121	0.126
Epicardial adipose tissue	WC	0.005	-0.042 to 0.053	0.828
SIVd	WC	0.009	0.005 to 0.013	**<0.001**
PPd	WC	0.005	0.001 to 0.009	**0.022**
DTD	WC	0.013	0.001 to 0.025	**0.031**
LVMi	WC	0.765	0.377 to 1.153	**<0.001**
RWT	WC	0.001	-0.001 to 0.003	0.299
Ejection Fraction	WC	0.037	-0.085 to 0.159	0.543
E_wave	FLI	-0.481	-0.980 to 0.018	0.058
A wave	FLI	0.873	-0.653 to 2.398	0.202
E/A	FLI	-0.003	-0.007 to 0.001	**0.099**
E deceleration time	FLI	0.334	-1.093 to 1.761	0.638
LAV	FLI	0.037	-0.230 to 303	0.782
LAVi	FLI	-0.021	-0.157 to 0.116	0.760
e′ septal	FLI	0.031	-0.016 to 0.078	0.189
e′ lateral	FLI	-0.005	-0.058 to 0.049	0.866
E/e′ ratio septal	FLI	-0.064	-0.016 to -0.012	**0.018**
E/e′ ratio lateral	FLI	-0.058	-0.108 to -0.007	**0.026**
Tricuspid regurgitation peak velocity	FLI	0.026	-0.049 to 0.101	0.480
Epicardial adipose tissue	FLI	0.002	-0.003 to 0.007	0.468
SIVd	FLI	0.005	0.002 to 0.009	**0.007**
PPd	FLI	0.001	-0.003 to 0.005	0.625
DTD	FLI	0.004	-0.007 to 0.016	0.436
LVMi	FLI	0.288	-0.069 to 0.644	0.110

RWT	FLI	0.000	-0.002 to 0.002	0.974
Ejection Fraction	FLI	0.001	-0.115 to 0.117	0.988
E wave	FIB4	1.609	0.583 to 2.636	**0.005**
A wave	FIB4	-4.228	-44.836 to 36.259	0.804
E/A	FIB4	0.008	-0.005 to 0.020	0.216
E deceleration time	FIB4	-0.366	-4.467 to 3.735	0.858
LAV	FIB4	-0.211	-1.036 to 0.613	0.609
LAVi	FIB4	-0.080	-0.474 to 0.315	0.687
e′ septal	FIB4	0.012	-0.128 to 0.152	0.862
e′ lateral	FIB4	0.004	-0.145 to 0.153	0.954
E/e′ ratio septal	FIB4	0.120	-0.042 to 0.281	0.143
E/e′ ratio lateral	FIB4	0.122	-0.023 to 0.267	0.097
Tricuspid regurgitation peak velocity	FIB4	-0.019	-0.183 to 0.145	0.821
Epicardial adipose tissue	FIB4	-0.008	-0.020 to 0.003	0.144
SIVd	FIB4	-0.012	-0.022 to -0.002	**0.025**
PPd	FIB4	0.000	-0.011 to 0.010	0.948
DTD	FIB4	0.017	-0.015 to 0.048	0.288
LVMi	FIB4	-0.116	-1.205 to 0.972	0.831
RWT	FIB4	-0.001	-0.007 to 0.004	0.580
Ejection Fraction	FIB4	-0.009	-0.323 to 0.304	0.953
E wave	CAP	0.014	-0.148 to 0.177	0.859
A wave	CAP	0.113	-0.088 to 0.313	0.239
E/A	CAP	0.000	-0.001 to 0.001	0.743
E deceleration time	CAP	0.156	-0.204 to 0.516	0.391
LAV	CAP	-0.009	-0.098 to 0.080	0.841
LAVi	CAP	-0.008	-0.051 to 0.036	0.725
e′ septal	CAP	-0.005	-0.018 to 0.009	0.499
e′ lateral	CAP	-0.005	-0.019 to 0.010	0.504
E/e′ ratio septal	CAP	0.000	-0.020 to 0.020	0.979
E/e′ ratio lateral	CAP	0.001	-0.025 to 0.028	0.926
Tricuspid regurgitation peak velocity	CAP	0.017	0.002 to 0.033	**0.031**
Epicardial adipose tissue	CAP	0.001	-0.009 to 0.012	0.787
SIVd	CAP	0.001	0.000 to 0.002	**0.022**
PPd	CAP	0.001	0.000 to 0.002	**0.008**
DTD	CAP	0.001	-0.002 to 0.003	0.613
LVMi	CAP	0.106	0.017 to 0.196	**0.021**
RWT	CAP	0.001	0.000 to 0.001	**0.020**
Ejection Fraction	CAP	0.018	-0.009 to 0.044	0.191
E wave	LSM	-1.267	-5.723 to 3.220	0.567
A wave	LSM	3.703	-3.742 to 11.148	0.294
E/A	LSM	0.001	-0.027 to 0.028	0.965
E deceleration time	LSM	2.985	-6.594 to 12.563	0.536
LAV	LSM	0.398	-1.932 to 2.729	0.734
LAVi	LSM	-0.007	-1.150 to 1.136	0.990
e′ septal	LSM	-0.118	-0.494 to 0.258	0.533
e′ lateral	LSM	-0.330	-0.708 to 0.048	0.085
E/e′ ratio septal	LSM	0.306	.0.248 to 0.860	0.274
E/e′ ratio lateral	LSM	0.403	-0.302 to 1.108	0.257
Tricuspid regurgitation peak velocity	LSM	0.010	-0.405 to 0.424	0.964
Epicardial adipose tissue	LSM	-0.134	-0.410 to 0.142	0.337
SIVd	LSM	0.016	-0.010 to 0.043	0.212
PPd	LSM	0.010	-0.015 to 0.034	0.433
DTD	LSM	0.009	-0.064 to 0.081	0.816
LVMi	LSM	1.362	-1.131 to 3.854	0.279
RWT	LSM	0.002	-0.010 to 0.014	0.750
Ejection Fraction	LSM	0.559	-0.137 to 1.254	0.114

Multivariable linear regression model. Abbreviations: 95% CI, 95% Confidence Intervals; A wave, Atrial contraction transmitral flow velocity; BMI, Body Mass Index; βadj, age- and gender-adjusted linear regression coefficient; CAP, Controlled Attenuation Parameter; DTD, Left ventricular end-diastolic diameter; E wave, Early diastolic transmitral flow velocity; E/A, Ratio of E wave to A wave velocity; E deceleration time, Early diastolic deceleration time; E/e′ ratio lateral, Ratio of E wave to lateral e′; E/e′ ratio septal, Ratio of E wave to septal e′; Ejection Fraction, Left ventricular ejection fraction; Epicardial adipose tissue, Epicardial fat thickness; e′ lateral, Lateral mitral annular early diastolic velocity; e′ septal, Septal mitral annular early diastolic velocity; FLI, Fatty Liver Index; LAV, Left atrial volume; LAVi, Left atrial volume index; LVMi, Left ventricular mass index; PPd, Posterior wall thickness in diastole; RWT, Relative wall thickness; SIVd, Interventricular septum thickness in diastole; Tricuspid regurgitation peak velocity, Peak velocity of tricuspid regurgitant jet. p values < 0.05 are shown in bold.

Table S2. Age- and gender- adjusted linear regression coefficients of associations between anthropometric measures and oxidative stress markers.

Independent Variable	Dependent Variable	B_{adj}	95% CI	p value
**WC**	**AOPP**	0.161	-0.307 to 0.629	0.494
**WC**	**SH**	-0.777	-1.613 to 0.058	0.068
**WC**	**Proteins**	-0.001	-0.008 to 0.007	0.849
**WC**	**SH/p**	-0.011	-0.024 to 0.002	0.101
**WC**	**dROMs**	1.405	0.143 to 2.666	**0.030**
**WC**	**Glutathione**	-0.016	-0.048 to 0.016	0.323
**WC**	**Cysteine**	0.236	-0.440 to 0.913	0.488
**WC**	**Cysteinylglycine**	-0.019	-0.125 to 0.087	0.718
**BMI**	**AOPP**	0.280	-0.898 to 1.458	0.637
**BMI**	**SH**	-2.681	-4.734 to -0.628	**0.011**
**BMI**	**Proteins**	0.009	-0.009 to 0.027	0.335
**BMI**	**SH/p**	-0.048	-0.080 to -0.017	**0.003**
**BMI**	**dROMs**	2.686	-0.535 to 5.906	0.101
**BMI**	**Glutathione**	-0.053	-0.133 to 0.028	0.194
**BMI**	**Cysteine**	0.971	-0.721 to 2.663	0.256
**BMI**	**Cysteinylglycine**	-0.133	-0.397 to 0.131	0.318

Multivariable linear regression model. Abbreviations: 95% CI, 95% Confidence Intervals; AOPP, advanced oxidation protein products; Badj, age- and gender-adjusted linear regression coefficient; BMI, Body Mass Index; dROMs, derivatives of reactive oxygen metabolites; SH/p, total free thiols normalized to protein content; WC, Waist Circumference. p values < 0.05 are shown in bold.

Table S3. Age- and gender-adjusted linear regression coefficients of associations between oxidative stress markers and steatosis and fibrosis parameters.

Independent Variable	Dependent Variable	B_{adj}	95% CI	p value
**AOPP**	**FLI**	0.272	-0.074 to 0.618	0.120
**SH**	**FLI**	0.044	-0.121 to 0.209	0.593
**Proteins**	**FLI**	-4.956	-22.729 to 12.818	0.576
**SH/p**	**FLI**	3.134	-6.152 to 12.420	0.499
**dROMs**	**FLI**	0.093	-0.017 to 0.203	0.096
**Glutathione**	**FLI**	-4.126	-8.071 to -0.182	**0.041**
**Cysteine**	**FLI**	-0.033	-0.209 to 0.142	0.705
**Cysteinylglycine**	**FLI**	0.251	-0.881 to 1.383	0.656
**AOPP**	**FIB4**	-0.032	-0.107 to 0.042	0.387
**SH**	**FIB4**	-0.004	-0.355 to 0.270	0.787
**Proteins**	**FIB4**	-1.005	-5.879 to 3.869	0.681
**SH/p**	**FIB4**	-0.047	-2.393 to 2.299	0.968
**dROMs**	**FIB4**	-0.004	-0.030 to 0.021	0.749

**Glutathione**	**FIB4**	0.302	-0.754 to 1.359	0.568
**Cysteine**	**FIB4**	-0.001	-0.050 to 0.049	0.975
**Cysteinylglycine**	**FIB4**	-0.028	-0.330 to 0.273	0.851
**AOPP**	**CAP**	-0.181	-0.742 to 0.381	0.552
**SH**	**CAP**	0.015	-0.294 to 0.324	0.924
**Proteins**	**CAP**	10.503	-25.867 to 46.873	0.556
**SH/p**	**CAP**	-2.917	-22.600 to 16.766	0.768
**dROMs**	**CAP**	0.141	-0.059 to 0.340	0.164
**Glutathione**	**CAP**	-1.933	-10.221 to 6.355	0.643
**Cysteine**	**CAP**	0.134	-0.253 to 0.522	0.491
**Cysteinylglycine**	**CAP**	0.674	-1.817 to 3.166	0.591
**AOPP**	**LSM**	0.014	-0.007 to 0.035	0.194
**SH**	**LSM**	-0.001	-0.013 to 0.011	0.848
**Proteins**	**LSM**	-0.034	-1.425 to 1.358	0.962
**SH/p**	**LSM**	-0.070	-0.822 to 0.682	0.853
**dROMs**	**LSM**	0.000	-0.007 to 0.008	0.934
**Glutathione**	**LSM**	-0.100	-0.412 to 0.212	0.524
**Cysteine**	**LSM**	-0.012	-0.027 to 0.003	0.106
**Cysteinylglycine**	**LSM**	0.078	-0.015 to 0.172	0.099

Multivariable linear regression model. Abbreviations: 95% CI, 95% Confidence Intervals; AOPP, advanced oxidation protein products; Badj, age- and gender-adjusted linear regression coefficient; CAP, Controlled Attenuation Parameter; dROMs, derivatives of reactive oxygen metabolites; FIB-4, fibrosis-4; FLI, Fatty Liver Index; SH/p, total free thiols normalized to protein content. p values < 0.05 are shown in bold.
